# Predicting high-risk return at emergency department presentation for patients who undergo short-term revisits: the HANDLE-24 score

**DOI:** 10.1186/s12873-025-01184-1

**Published:** 2025-02-13

**Authors:** Chung-Ting Chen, Po-Hsiang Liao, Meng-Chen Lin, Hsien-Hao Huang, Chorng-Kuang How, Yu-Chi Tung

**Affiliations:** 1 Emergency Department, Taipei Veterans General Hospital, Taipei, Taiwan; 2 School of Medicine, National Yang Ming Chiao Tung University, Taipei, 112 Taiwan; 3 Institute of Health Policy and Management, College of Public Health, National Taiwan University, No.17, Xu-Zhou Rd, Taipei, 10055 Taiwan; 4 Institute of Public Health, School of Medicine, National Yang Ming Chiao Tung University, Taipei, 112 Taiwan; 5 Nursing Department, Taipei Veterans General Hospital, Taipei, Taiwan; 6 Institute of Emergency and Critical Care Medicine, National Yang Ming Chiao Tung University, Taipei, Taiwan; 7 Master of Public Health Program, College of Public Health, National Taiwan University, No.17, Xu-Zhou Rd, Taipei, 10055 Taiwan

**Keywords:** Emergency department revisit, Emergency department return, Mortality, Prediction model, Risk score, Quality measure

## Abstract

**Background:**

The 72-h emergency department (ED) revisit rate is a key quality indicator. While some revisits stem from medical errors or inadequate initial treatment, others are due to disease progression or a lack of accessible care. The development of a risk assessment tool could identify high-risk patients and improve resource management.

**Methods:**

This study was conducted via an electronic health records system at a tertiary center in Taiwan. We derived a risk model via logistic regression and bootstrapping methods using a retrospective cohort of adults who underwent 72-h ED revisits between January 2019 and December 2020. The study population was divided into development (2019: 1224) and validation datasets (2020: 985). The primary outcome was high-risk return, defined as intensive care unit (ICU) admission or in-hospital mortality after 72-h ED return.

**Results:**

On the basis of the odds ratio, eight variables were independently associated with high-risk ED returns and subsequently included in the HANDLE-24 score (hypertension; symptoms of acute coronary syndrome; dysnatremia; dyspnea; liver disease; triage level escalation; and revisits within 24 h). The area under the receiver operating characteristic curve was 0.816 (95% CI: 0.760–0.871, p < 0.001) in the development dataset and 0.804 (0.750–0.858) in the validation dataset. Patients can be divided into three risk categories on the basis of the HANDLE-24 score: low [0–8.5], moderate [9–11.5] and high [12–22] risk groups. The ability of our risk score to predict the rates of hospital admission, ICU admission and in-hospital mortality was significant according to the Cochran‒Armitage trend test.

**Conclusion:**

The HANDLE-24 score represents a simple tool that allows early risk stratification and suggests more aggressive therapeutic strategies for patients experiencing ED revisits. The risk of adverse outcomes in ED adults after revisiting can be swiftly assessed via easily available information.

## Introduction

The emergency department (ED) revisits within 72 h of discharge is generally recognized as a key indicator of the quality of ED care [1–4]. As a benchmark to improve the health care quality of ED services and work efficiency and to ensure patient safety [5, 6], the use of 72-h ED returns as a measure of interest derives from the commonly held belief that these patients represent premature discharges from the first ED visit and may be associated with unsafe or ineffective care. Although previous studies have identified risk factors for ED return [7, 8], limited data are available that focus on the subsequent outcomes of ED revisits. ED revisits can be attributed not only to medical errors and inadequate diagnoses or treatment during the initial visit but also to the nature of the disease itself. Some patients discharged from the ED may be underserved due to a lack of accessible local primary care and/or specialty services [9], whereas others could return for nonemergency problems [10].

ED revisit within 72 h seems to be inevitable and may not be absolutely due to quality of care issues but may result from the natural progression of disease or other intricate factors. Therefore, we aimed to develop a risk assessment tool for ED returns with poor outcomes, which can allow early identification of high-risk patients who might require personalized local care and/or specialty and some targeted interventions. Predicting which ED revisiting patients are likely to experience adverse outcomes would help in planning and adequately managing resources.

## Methods

### Study design and setting

This study was a retrospective cohort analysis based on data collected between January 2019 and December 2020 from electronic health record (EHR) data from a tertiary academic medical center in Taiwan, Taipei Veteran General Hospital (TVGH). Patients had to meet the following inclusion criteria: individuals aged 18 years and above underwent any subsequent ED revisits within 72 h after an index treat-and-release ED visit discharge [1, 2, 11]. Our study targeted patients with the following exclusions: (1) patients with missing data; (2) patients who were against medical advice; and (3) patients who were frequent visitors to 5 or more EDs in the previous year. The study population was divided into two parts: the development cohort (2019) and the validation cohort (2020).

This study was approved by the Ethics Committee/Institutional Review Board of TVGH (Protocol Number: 2021–06-027CC), which waived the requirement for informed patient consent because of the retrospective nature of the analysis. Medical records were first reviewed by an emergency physician, followed by monitoring the subsequent chart review and data analysis by the corresponding author, who is also an emergency medicine specialist. We undergo regular training on research ethics and data collection in accordance with established standards.

### Data collection and outcomes

Routine patient demographic characteristics, past medical history, physiologic measures, and patient outcomes were retrospectively extracted and collected from the EHR system. Details of comorbidities, including history of malignancy, coronary artery disease, congestive heart failure, arrhythmia, hypertension, chronic lung disease, ischemic or hemorrhagic stroke, dementia, chronic kidney disease, diabetes, chronic liver disease, or peptic ulcer disease, were collected from the charts of the identified patients. Symptomatic tachycardia was defined as a heart rate > 150 beats per minute and < 50 beats per minute for symptomatic bradycardia. Dyspnea was defined as a respiratory rate > 20 or < 10 breaths per minute. The change in the Glasgow Coma Scale (GCS) score was computed as the difference between the return and index visits, and an exacerbation in the GCS score with a negative value suggested deterioration of the patient’s consciousness level. Similarly, the change in triage score was calculated by subtracting the score from two visits. A negative value indicating an escalation in triage level suggested an exacerbation in the patient's general health status. The blood tests included tests for white blood cell (WBC) counts, neutrophil counts, lymphocyte counts, hemoglobin levels, platelet counts, and sodium and potassium levels. The neutrophil-to-lymphocyte ratio (NLR), platelet-to-lymphocyte ratio (PLR), and systemic immune inflammation index (SII) were analyzed. We calculated the NLR, PLR, and SII as follows: NLR = neutrophil count/lymphocyte count, PLR = platelet count/lymphocyte count, and SII = platelet count × neutrophil count/lymphocyte count. Anemia was defined as a hemoglobin (Hb) level < 12.0 g/dL in women and < 13.0 g/dL in men. Thrombocytopenia was defined as a platelet count below the lower limit of normal (150,000/microliter). Hypernatremia and hyponatremia were defined as a serum sodium level greater than 145 mmol/L and less than 135 mmol/L, respectively. Hyperkalemia and hypokalemia were defined as a serum potassium level greater than 5.0 mmol/L and less than 3.5 mmol/L, respectively. The primary outcome was high-risk ED return, which was defined as subsequent intensive care unit (ICU) admission or in-hospital mortality after a 72-h ED revisit. The methodology of this study is consistent with the STROBE checklist for observational studies.

### Statistical analysis

Continuous variables are summarized as medians with interquartile ranges or means with standard deviations. Categorical data are expressed as frequencies and proportions. Continuous variables were assessed via the Mann‒Whitney U test for independent samples. Analysis of categorical variables was performed via Pearson’s chi-square test or Fisher’s exact test, as appropriate.

We set up a model from the development dataset (patient data from 2019) via multivariate analysis (including factors that were found to be significant with p < 0.10 in the univariate analysis), performing backward stepwise logistic regression analysis. Odds ratios (ORs) with 95% confidence intervals (CIs) are presented. The ORs from the logistic regression analysis were used to quantify the strength of the association between each variable and the high-risk outcomes. The measured OR in this multivariate analysis was used to assign points to the relevant parameters, giving a 0.5 score to each 0.5 value of OR, ensuring the weighting of predictors reflecting their relative influence on high-risk ED returns. We then establish a scoring system based on the variables independently associated with high-risk returns. Furthermore, external validation was carried out with patient data from 2020. The predictive ability of the final score (discrimination) was assessed via receiver operating characteristic (ROC) curve analysis in the development and validation datasets. Areas under the ROC curves (AUCs, or Harrell's C statistics) are presented with 95% CIs for both the development and the validation cohorts. The ROC curves illustrate the degree to which the created scoring system could discriminate high-risk ED revisits from non-high-risk ones by a graphical illustration of the trade-offs between sensitivity and specificity at each cutoff. The best cutoff point would be located at the upper left corner (100% sensitivity and 100% specificity) [12]. To assess the calibration, Hosmer–Lemeshow goodness-of-fit tests using deciles of high-risk returns were performed on the development and validation cohorts and on the 2000 bootstrapping resamples. All analyses were processed via IBM SPSS Statistics software (version 20.0; IBM Corp., Armonk, NY, USA). All tests were 2-tailed, and a p value < 0.05 was considered statistically significant.

## Results

**Fig. 1:**
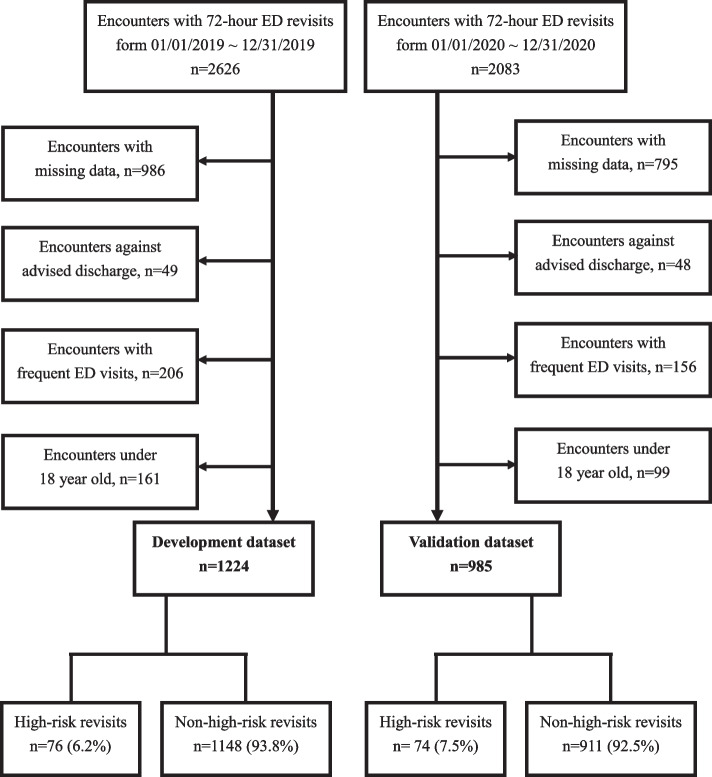
Flow chart of the development and validation datasets

During the study period, 1224 and 985 encounters were identified as eligible patients who experienced 72 h of ED revisits for the development and validation datasets, respectively (Fig. 1). The baseline characteristics and outcomes of the development and validation datasets are illustrated in Table 1. In the development dataset, 5.1% (*n* = 63) experienced ICU admission, 2.2% (*n* = 27) experienced in-hospital mortality, 41.4% (*n* = 507) experienced hospital admission, and 0.2% (*n* = 2) revisited as OHCAs. In comparison, 4.7% (*n* = 46) experienced ICU admission, 3.9% (*n* = 38) experienced in-hospital mortality, 48.9% (*n* = 482) experienced hospital admission, and 0.1% (*n* = 1) revisited OHCAs in the validation dataset. Patient characteristics for the development dataset are summarized in Table 2 and are subdivided into high-risk and non-high-risk revisits. Encounters with high-risk revisits were older, were more likely to be hospitalized within 3 months, had a higher incidence of mean arterial pressure (MAP) less than 65 mmHg, dyspnea, deterioration of GCS, escalation of triage level when revisiting, interval of two ED visits ≤ 24 h, and length of stay of index visit ≥ 240 min. Individuals with high-risk returns had a greater incidence of comorbidities such as coronary artery disease, congestive heart failure, hypertension, chronic lung disease, stroke, chronic kidney disease, diabetes mellitus, and chronic liver disease than did patients with non-high-risk returns. Patients who experienced adverse outcomes after ED revisiting were more likely to complain of acute coronary syndrome (ACS)-related symptoms, such as chest tightness or chest discomfort. High-risk returns were significantly associated with a high NLR and a high incidence of anemia, thrombocytopenia, hypernatremia, and hyponatremia.

**Table 1:** Characteristics and outcomes of development and validation dataset

			Development dataset (*N* = 1224)	Validation dataset (*N* = 985)
Age, mean(S.D.)			64.3(19.9)	62.4(20.4)
Male, N(%)			641(52.4)	528(53.6)
Recent hospitalization, N(%)			281(23.8)	244(24.8)
Vital signs when revisiting, mean(S.D.)				
SBP (mmHg)			141(30)	141(29)
DBP (mmHg)			78(15)	76(15)
Body temperature (℃)			36.7(0.9)	36.7(1.0)
Heart rate (/min)			89(20)	89(20)
Respiratory rate (/min)			20(2.7)	20(2.3)
Escalation of triage level, N(%)			194(15.8)	140(14.2)
Interval between two ED visits(hrs)				
Mean(S.D.)			41.2(17.1)	40.0(17.0)
LOS of index ED (mins)				
Mean(S.D.)			581(856)	465(589)
LOS of revisit ED (mins)				
Mean(S.D.)			968(1175)	845(1065)
Outcomes after revisiting				
High-risk revisits, N(%)			76(6.2)	74(7.5)
ICU admission, N(%)			63(5.1)	46(4.7)
In-hospital mortality, N(%)			27(2.2)	38(3.9)
Hospital admission, N(%)			507(41.4)	482(48.9)
OHCA, N(%)			2(0.2)	1(0.1)
Hospital LOS, median(IQR)			8(5–15)	9(6–14)

*S.D* Standard deviation, Recent hospitalization: hospitalization within 3 months, *SBP* Systolic blood pressure, *DBP* Diastolic blood pressure, *ED* Emergency department, *LOS* Length of stay, *ICU* Intensive care unit, Hospital admission: any hospital admission regardless of ICU admissions and in-hospital mortality, *OHCA* Out-of-hospital cardiac arrest, All OHCA patients were admitted to ICU, *IQR* Interquartile range

**Table 2:** Comparison of high-risk and non-high-risk revisits of development dataset

(*N* = 1224)	High-risk revisits ss(*N* = 76)	Non-high-risk revisits (*N* = 1148)	*p* value
Age, mean(S.D.)	73.7(17.1)	63.7(19.9)	< 0.001*
Male, N(%)	43(56.6)	598(52.1)	0.448
Recent hospitalization, N(%)	29(38.2)	262(22.8)	0.002*
Vital signs when revisiting			
MAP < 65 mmHg, N(%)	7(9.2)	21(1.8)	< 0.001*
Dyspnea, N(%)	33(43.4)	185(16.1)	< 0.001*
GCS deterioration, N(%)	16(21.1)	40(3.5)	< 0.001*
Escalation of triage level, N(%)	44(57.9)	150(13.1)	< 0.001*
Interval of two visits ≤ 24 h, N(%)	24(31.6)	203(17.7)	0.003*
LOS of index ED ≥ 240 min, N(%)	47(61.8)	567(49.4)	0.036*
Comorbidities, N(%)			
Malignancy	18(23.7)	196(17.1)	0.142
Coronary artery disease	20(26.3)	189(16.5)	0.027*
Congestive heart failure	12(15.8)	61(5.3)	< 0.001*
Hypertension	52(68.4)	488(42.5)	< 0.001*
Chronic lung disease	7(9.2)	46(4.0)	0.031*
Stroke	13(17.1)	70(6.1)	< 0.001*
Chronic kidney disease	9(11.8)	69(6.0)	0.044*
Diabetes mellitus	28(36.8)	257(22.4)	0.004*
Chronic liver disease	11(14.5)	79(6.9)	0.014*
Chief complaints, N(%)			
Chest discomfort	11(14.5)	85(7.4)	0.026*
Shortness of breath	7(9.2)	56(4.9)	0.098
Focal weakness	2(2.6)	9(0.8)	0.098
Laboratory finding, N(%)			
WBC > 12,000 or < 4000 (× 10^3^/ul)	22(28.9)	243(21.2)	0.111
NLR	10.3(12.1)	6.8(9.3)	< 0.001*
PLR	256(255)	228(241)	0.379
SII	1819(2234)	1484(2367)	0.066
Anemia	42(55.3)	461(40.2)	0.010*
Thrombocytopenia	26(34.2)	215(18.7)	0.001*
Hypernatremia	9(11.8)	25(2.2)	< 0.001*
Hyponatremia	19(25.0)	163(14.2)	0.010*
Hyperkalemia	6(7.9)	46(4.0)	0.104
Hypokalemia	9(11.8)	129(11.2)	0.872

*S.D* Standard deviation, *MAP* Mean arterial pressure, *GCS* Glasgow Coma Scale, *LOS* Length of stay, *ED* Emergency department, *NLR* Neutrophil-to-lymphocyte ratio, *PLR* Platelet-to-lymphocyte ratio, *SII* Systemic immune inflammation index

^*^p < 0.05

**Fig. 2:**
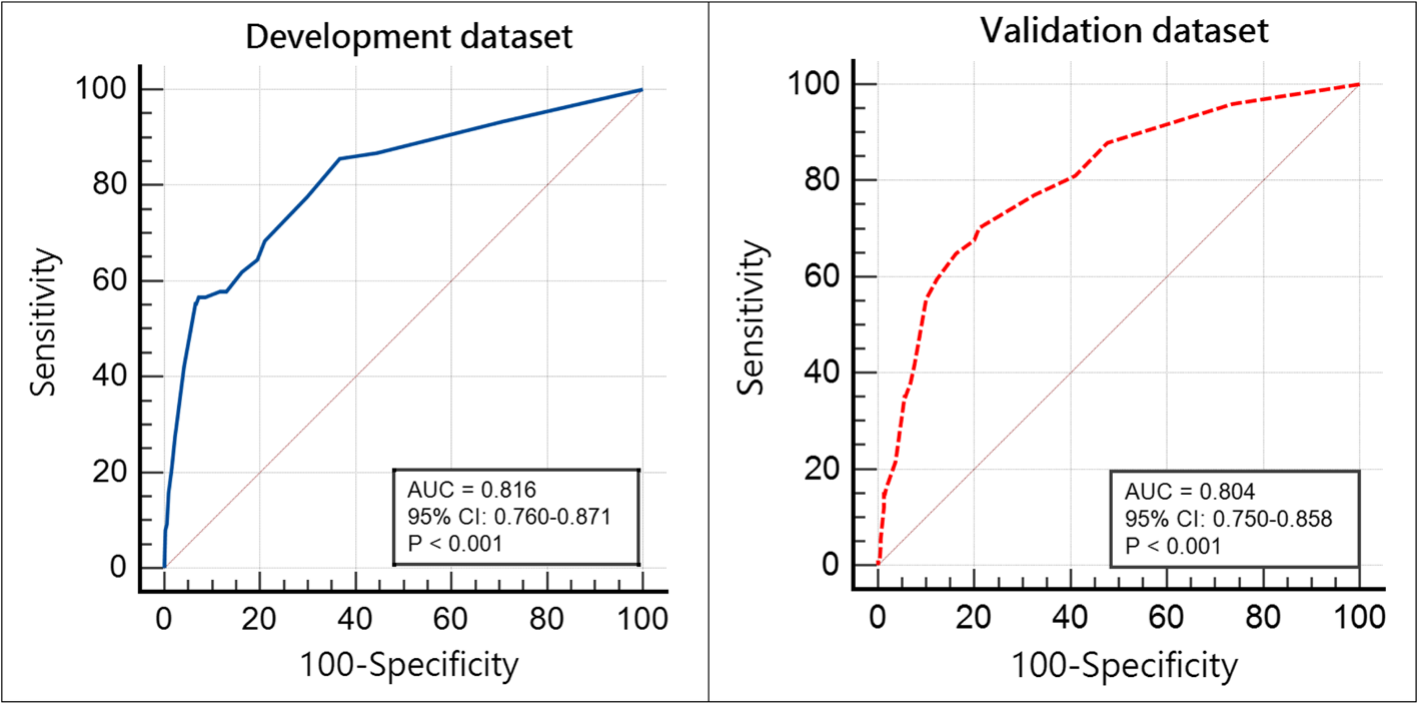
Receiver operating characteristic curves for high-risk ED return in the development and validation datasets

Multivariate analysis revealed that dyspnea, escalation of triage level, interval of two visits ≤ 24 h, comorbidities of hypertension and chronic liver disease, complaint of chest discomfort, and revisiting with hypernatremia and hyponatremia were identified as independent predictors for high-risk returns (Table 3). The total of these eight factors established a fresh scoring system with a metric called the HANDLE-24 score, ranging from 0–22. The HANDLE-24 score consists of *hypertension, acute coronary syndrome (ACS) symptoms, dysnatraemia, dyspnea, chronic liver disease, an increase in the triage level,* and *revisits within 24 h* (Table 4). A HANDLE-24 score at a threshold of 8.5 was used to predict a high-risk return; the sensitivity was 55.3%, and the specificity was 92.7%, with a positive likelihood ratio = 7.577 and a negative likelihood ratio = 0.482, indicating that moderate evidence exists. The AUCs of the HANDLE-24 score in the development and validation datasets were 0.816 (95% CI 0.760–0.871, *P* < 0.001) and 0.804 (95% CI 0.750–0.858, *P* < 0.001), respectively (Fig. 2), indicating good diagnostic accuracy, and the Hosmer–Lemeshow goodness-of-fit tests were chi-square = 7.417 (*p* = 0.284) and 1.743 (*p* = 0.942), respectively, indicating that the logistic model was appropriate for our analysis. The adults who underwent 72 h ED visits were categorized into three groups according to their HANDLE-24 scores (low-risk group: 0–8.5, moderate-risk group: 9–11.5, and high-risk group: 12–22). The probabilities of adverse outcomes with subsequent ICU admission or in-hospital mortality after ED revisiting for the low-, moderate-, and high-risk groups were 3.0%, 27.5%, and 44.7%, respectively; the differences between these rates were statistically significant (Cochran-Armitage trend test, *p* = 0.001) (Table 5). When hospital admission, ICU admission and in-hospital mortality were investigated, the newly developed scoring system was also able to predict outcomes significantly.

**Table 3:** Logistic regression analysis of predictors of the high-risk revisit among encounters with 72-h ED revisits

(*N* = 1224)	Univariate analysis		Multivariate analysis	
	Odds ratio (95% CI)	*p* value	Odds ratio (95% CI)	*p* value
Age	1.029 (1.015–1.043)	< 0.001*		
Recent hospitalization	2.087 (1.287–3.382)	0.003*		
Revisit MAP < 65 mmHg	5.444 (2.237–13.25)	< 0.001*		
**Dyspnea**	3.995 (2.472–6.457)	< 0.001*	2.104 (1.225–3.613)	0.007*
GCS deterioration	7.387 (3.913–13.94)	< 0.001*		
**Escalation of triage level**	9.148 (5.623–14.88)	< 0.001*	6.182 (3.649–10.47)	< 0.001*
**Interval of 2 visits ≤ 24 h**	2.149 (1.294–3.567)	0.003*	1.856 (1.059–3.255)	0.031*
LOS of index visiting ≥ 4 h	1.661 (1.031–2.676)	0.037*		
Coronary artery disease	1.812 (1.063–3.091)	0.029*		
Congestive heart failure	3.341 (1.713–6.519)	< 0.001*		
**Hypertension**	2.930 (1.782–4.820)	< 0.001*	1.933 (1.126–3.317)	0.017*
Chronic lung disease	2.430 (1.058–5.582)	0.036*		
Stroke	3.178 (1.669–6.052)	< 0.001*		
Chronic kidney disease	2.101 (1.005–4.390)	0.048*		
Diabetes mellitus	2.022 (1.244–3.289)	0.005*		
**Chronic liver disease**	2.290 (1.162–4.514)	0.017*	2.477 (1.153–5.320)	0.020*
**Complaint of chest tightness**	2.116 (1.076–4.161)	0.030*	2.642 (1.251–5.577)	0.011*
Revisit NLR	1.023 (1.007–1.039)	0.005*		
**Revisit hypernatremia**	6.034 (2.709–13.44)	< 0.001*	4.833 (1.915–12.19)	0.001*
**Revisit hyponatremia**	2.014 (1.168–3.474)	0.012*	2.320 (1.260–4.273)	0.007*
Anemia	1.841 (1.154–2.937)	0.010*		
Thrombocytopenia	2.257 (1.373–3.708)	0.001*		

*ED* Emergency department, *MAP* Mean arterial pressure, *GCS* Glasgow Coma Scale, *LOS* Length of stay, *NLR* Neutrophil-to-lymphocyte ratio

In the univariate analysis, only the variables with *p* < 0.10 were listed

^*^p < 0.05

**Table 4:** Point allocation for predictors of the high-risk ED revisit in the development dataset as HANDLE-24 score

Predictive factor	Odd ratio ( 95% CI)	Score assigned
Hypertension	1.933 (1.126–3.317)	2.0
Complaint of chest discomfort/tightness	2.642 (1.251–5.577)	2.5
Revisit hypernatremia	4.833 (1.915–12.19)	5.0
Revisit hyponatremia	2.320 (1.260–4.273)	2.5
Dyspnea (RR > 20 or < 10 /mins)	2.104 (1.225–3.613)	2.0
Chronic liver disease	2.477 (1.153–5.320)	2.5
Escalation of triage level	6.182 (3.649–10.47)	6.0
Interval of two visits ≤ 24 h	1.856 (1.059–3.255)	2.0

The measured odd ratios from the multivariate analysis were used to assign points to the relevant parameters, giving a score of 0.5 for every OR value of around 0.5, ensuring the weighting of predictors reflecting their relative influence on high-risk ED returns

*CI* Confidence interval, *H* Hypertension, *A* ACS symptoms, *N* dysnatremia, *D* Dyspnea, *L* Liver disease, *E* Escalation of triage level, *24* revisits within 24 h

**Table 5:** Distribution of outcomes among adult patients with ED returns regarding HANDLE-24 score

Risk category	High-risk return	Hospital admission	ICU admission	In-hospital mortality
Low (0 ~ 8.5)	3.0%	38.1%	2.2%	1.2%
Moderate (9 ~ 11.5)	27.5%	66.3%	26.3%	6.3%
High (12 ~ 22)	44.7%	76.6%	38.3%	19.1%
Cochran-Armitage trend test	< 0.001*	< 0.001*	< 0.001*	< 0.001*

*ED* Emergency department, *ICU* Intensive care unit

^*^p < 0.05

## Discussion

We conducted this investigation to report the characteristics of ED 72-h revisits as well as the subsequent outcomes. Our study revealed that high-risk ED returns were significantly associated with hypertension, chest tightness or discomfort symptoms, hypernatremia, hyponatremia, a respiratory rate > 20 or < 10 breaths per minute, chronic liver disease, an escalation of the triage level, and a short revisiting interval within 24 h. On the basis of the materials explored in this study, we propose a simple and practical risk score that can be readily applied by clinicians to evaluate individual risk of poor outcomes after revisiting. By systematically addressing these predictors, healthcare providers can significantly improve the prognosis for patients with short-term ED revisits. The predictors included in the risk prediction score are as follows: 1) preexisting characteristics (hypertension and chronic liver disease); 2) subjective ED utilization pattern (interval of two ED visits ≤ 24 h) and presenting symptoms (ACS symptoms); and 3) objective signs (dyspnea), laboratory abnormalities (dysnatremia) and clinical assessments (escalation of triage level). The ability of the risk score to predict the rates of adverse outcomes of subsequent ICU admission or in-hospital mortality was further evaluated separately according to different risk groups in the development and validation sets. Significant increases in the rates of adverse outcomes were observed with increasing risk scores in both sets of patients. Our ED risk stratification model offers new opportunities to test coordinated care strategies that address multiple conditions across various demographic groups, which we believe may improve case management effectiveness.

The ROC curve is a graphical representation of a model's ability to distinguish between different outcomes—in our case, high-risk versus non-high-risk ED revisits. The AUC quantifies the overall performance of the prediction model, with values ranging from 0.5 (no discrimination) to 1.0 (perfect discrimination). An AUC of ≥ 0.70 is defined as a clinically useful discriminative ability [13]. An AUC of 0.816 in our development dataset and 0.804 in the validation dataset indicates that the HANDLE-24 score has good discriminative ability. To integrate the HANDLE-24 score into routine practice, ED systems can embed this model into EHRs, automating the risk calculation right after laboratory data is available for ED revisiting patients. Real-time AUC-derived probabilities can guide immediate clinical decisions, ensuring that care is targeted efficiently and equitably.

The HANDLE-24 score was developed and validated using data from a single tertiary academic medical center. While the external validation cohort from 2020 confirms its robustness within this setting, the model's applicability to other hospital environments or patient populations warrants further discussion. Despite challenges, the HANDLE-24 score has key strengths that support its potential for generalizability: The model relies on readily available clinical and laboratory data, making it feasible to implement across a wide range of hospitals; the stratification into risk groups aligns well with universal clinical decision-making processes, enhancing its applicability. Indeed, further research is still needed to validate the HANDLE-24 score in different hospital types, geographic regions, and patient populations. Such studies will provide insights into the score's adaptability and ensure its utility in enhancing patient care and resource allocation across diverse healthcare systems.

When patients return to the ED after discharge, it is generally believed that these revisits are due to medical errors, inadequacies in the initial evaluation or treatment, or the nature of the disease [10]. Specifically, a revisit is not merely a reflection of the care received during the initial visit; it may result from disease progression despite appropriate care or from a lack of access to necessary outpatient care. Although ED revisits within 72 h are widely regarded as indicators of the quality of care in the ED, their impact on patient outcomes has not been proven and may not accurately reflect the actual quality of care received. They may instead reflect different opportunities to access primary care or community-based care [14]. Several studies have shown that patients prefer to seek care in the ED rather than in the outpatient setting and that a patient returning to the ED is not inherently an adverse event [15–17]. In some guidelines, however, expert consensus recommends the use of unscheduled return visits with admission to monitor ED performance [18]. Since ED revisits may not be entirely preventable, it is crucial to concentrate on distinguishing risk levels when these revisits do occur. Identifying risk factors and effective prevention strategies are essential for alleviating the burden on the ED healthcare system. The ability to accurately identify which patients are more likely to experience adverse outcomes when revisiting could improve treatment plans and disposition decisions and allow EDs and health systems to develop more focused interventions. Our goal, therefore, is not to entirely eliminate ED revisits but rather to help foster and develop a system by which we can more reliably identify those revisits occurring due to potential lapses in quality or care systems so that we can better address the underlying quality issues.

Electrolyte disturbances are among the most commonly investigated abnormalities among laboratory studies of ED patients in clinical practice. They are crucial indicators of an ill patient's clinical status, frequently leading to adjustments in their treatment plan. Hypo- and hypernatremia are common electrolyte abnormalities that lead to a spectrum of clinical symptoms [19]. An association between dysnatremia and adverse outcomes has been reported across a wide range of diseases (e.g., cardiac arrest, acute myocardial infarction, heart failure, cirrhosis, sepsis, pneumonia, pulmonary embolism and cancer) [20–27]. In our study, both hypo- and hypernatremia, when revisiting, were significantly associated with subsequent adverse outcomes. Abnormalities in serum sodium levels adversely affect pivotal physiological parameters such as intracellular hydration [28] and increase both morbidity and mortality [19]. Indeed, the causes of dysnatremia may also affect outcomes. Thus, it remains to be determined whether dysnatremia directly contributes to adverse outcomes or is a manifestation of severe tissue damage. Given the variety of complex and diverse conditions that ED physicians must face, routine monitoring of serum sodium should be a reliable general assessment tool.

To the best of our knowledge, this is the first study attempting to develop a risk scoring system to predict adverse outcomes for individuals undergoing short-term ED revisiting on the basis of a retrospective cohort. Retrospective use cases are extremely valuable, offering more precise risk adjustment in observational research and enhancing quality improvement efforts related to ED capacity strain, triage processes, and patient flow management. The application of such a predictive tool, the HANDLE-24 score, in clinical practice provides a multitude of advantages, including early identification of high-risk ED revisiting patients, tailored treatment plans, improved monitoring and follow-up, optimized resource utilization, informed clinical decision-making, enhanced patient education and engagement, and data-driven improvements in care quality. The HANDLE-24 score demonstrates robust predictive ability for high-risk ED revisits in a single-center study. Its application may aid clinicians in identifying patients who require closer monitoring or early intervention, but further validation in diverse settings is still necessary.

## Limitations

Our study has a few limitations, even though we attempted to compensate for all the possible biases by adjusting for all known confounders that may have influenced our results in a multivariate regression model. First, this study was a retrospective analysis of clinical data, which included detailed demographic, hospital, physiological, and laboratory information. There may have been confounding factors affecting the relationship between variables and outcomes that were not considered in this analysis. The retrospective design inherently introduced potential biases. The reliance on existing EHR may result in incomplete or inconsistent data points, particularly for unmeasured variables. Additionally, retrospective analyses did not allow for controlling variables prospectively, which may affect the robustness of the results. Second, we did not collect the following data on a correction but, instead, considered only laboratory findings on ED returns. Whether laboratory-level trajectories play a role requires further elucidation in the future. Furthermore, our data were derived from a single center, and the results from this single-site study cannot necessarily be generalized to disparate clinical settings. Finally, assessing chief complaints during return visits could provide valuable insights into symptom progression and the reasons for revisits. However, we didn't collect the data for the chief complaint at the return visit.

## Conclusions

In conclusion, we developed the HANDLE-24 score using both administrative data and laboratory studies collected shortly after an ED returned (generally within 1 h) to predict adverse outcomes among all revisiting adults, which has been further validated. Our study suggests that this HANDLE-24 score is successful in distinguishing adult patients undergoing 72-h ED revisiting into three different risk groups, including those with subsequent hospital admission, ICU admission, or in-hospital mortality. The HANDLE-24 score provides a simple, validated tool for predicting adverse outcomes following ED revisits within 72 h. By integrating routinely available clinical and laboratory data, this model can assist in early risk stratification. However, its applicability to broader patient populations and diverse healthcare settings requires further prospective, multicenter studies. Addressing these gaps will enhance its utility in improving patient outcomes and optimizing ED resource allocation.

## Data Availability

The datasets generated and analyzed during the current study are not publicly available but are available from the corresponding author upon reasonable request.

## Abbreviations

ACS: Acute coronary syndrome
AUC: Areas under the curve
CI: Confidence intervals
ED: Emergency department
GCS: Glasgow Coma Scale
Hb: Hemoglobin
ICU: Intensive care unit
NLR: Neutrophil to lymphocyte ratio
OHCA: Out-of-hospital cardiac arrests
OR: Odds ratio
PLR: Platelet to lymphocyte
ROC: Receiver operating characteristic
SII: Systemic immune inflammation index
TVGH: Taipei Veteran General Hospital
WBC: White blood cell
